# Histone H3 Cleavage in Severe COVID-19 ICU Patients

**DOI:** 10.3389/fcimb.2021.694186

**Published:** 2021-09-10

**Authors:** Joram Huckriede, Femke de Vries, Michael Hultström, Kanin Wichapong, Chris Reutelingsperger, Miklos Lipcsey, Pablo Garcia de Frutos, Robert Frithiof, Gerry A. F. Nicolaes

**Affiliations:** ^1^Department of Biochemistry, Cardiovascular Research Institute Maastricht (CARIM), Maastricht University, Maastricht, Netherlands; ^2^Department of Surgical Sciences, Section for Anaesthesia & Intensive Care, Uppsala University, Uppsala, Sweden; ^3^Department of Medical Cell Biology, Integrative Physiology, Uppsala University, Uppsala, Sweden; ^4^Hedenstierna Laboratory, Anaesthesiology and Intensive Care Medicine, Department of Surgical Sciences, Uppsala University, Uppsala, Sweden; ^5^Department of Cell Death and Proliferation, IIBB-CSIC, IDIBAPS and CIBERCV, Barcelona, Spain

**Keywords:** extracellular histones, DAMPs (damage-associated molecular patterns), COVID-19, NETosis, ICU - intensive care unit

## Abstract

The severity of coronavirus disease 19 (COVID-19) is associated with neutrophil extracellular trap (NET) formation. During NET formation, cytotoxic extracellular histones are released, the presence of which is linked to the initiation and progression of several acute inflammatory diseases. Here we study the presence and evolution of extracellular histone H3 and several other neutrophil-related molecules and damage-associated molecular patterns (DAMPs) in the plasma of 117 COVID-19-positive ICU patients. We demonstrate that at ICU admission the levels of histone H3, MPO, and DNA-MPO complex were all significantly increased in COVID-19-positive patients compared to control samples. Furthermore, in a subset of 54 patients, the levels of each marker remained increased after 4+ days compared to admission. Histone H3 was found in 28% of the patients on admission to the ICU and in 50% of the patients during their stay at the ICU. Notably, in 47% of histone-positive patients, we observed proteolysis of histone in their plasma. The overall presence of histone H3 during ICU stay was associated with thromboembolic events and secondary infection, and non-cleaved histone H3 was associated with the need for vasoactive treatment, invasive ventilation, and the development of acute kidney injury. Our data support the validity of treatments that aim to reduce NET formation and additionally underscore that more targeted therapies focused on the neutralization of histones should be considered as treatment options for severe COVID-19 patients.

## Introduction

The global pandemic of the severe acute respiratory syndrome coronavirus (SARS-CoV-2) has infected over 120 million people and caused 2.5 million deaths as of March 2021 to the associated coronavirus disease 19 (COVID-19) (WHO, 2021). For many patients, COVID-19 appears to be an asymptomatic infection, but about 20% of the patients develop mild clinical symptoms like a common cold. The more severe cases usually develop bilateral interstitial pneumonia and moderate to severe hypoxemia, resulting in respiratory failure and acute respiratory distress syndrome (ARDS) (Zhou et al., 2020). Recent data show that the most serious syndrome is associated with early localized proinflammatory cytokine release (Wang et al., 2020), hypercoagulability (Middleton et al., 2020; Voicu et al., 2020), and neutrophil extracellular trap (NET) formation (Zuo et al., 2020; Busch et al., 2020).

As the SARS-CoV-2 viral infection activates neutrophils to form NETs (Busch et al., 2020; Veras et al., 2020), their intracellular components are released into the extracellular space. These intracellular components include cell-free DNA (cfDNA), histones, and granules containing neutrophil elastase (NE) and myeloperoxidase (MPO) (Brinkmann et al., 2004). Released NE and MPO both function as antimicrobial factors, as NE is a serine protease able to degrade virulence factors and kill bacteria, and MPO catalyzes the formation of the antimicrobial hydrogen peroxide (Papayannopoulos et al., 2010). The majority, around 70% of the NETs proteins, consists of extracellular histones. Histones are normally responsible for packaging DNA into nucleosomes inside the nucleus of eukaryotic cells. Two copies of histone H2A, H2B, H3, and H4 constitute the core of nucleosomes, around which 146 double helix DNA base-pairs are wound (Brinkmann et al., 2004). The release of histones during NET formation or after cell death facilitates the histone-mediated neutralization of pathogens (Silvestre-Roig et al., 2019). However, extensive extracellular levels of histone cause detrimental effects for the host (Silk et al., 2017). Extracellular histones act as damage-associated molecular patterns (DAMPs) that contribute to the immune response by promoting immune cell activation, inflammasome formation, and proinflammatory cytokine release. Yet, extracellular histones are able to damage a range of host cells (Xu et al., 2009; Abrams et al., 2013; Cheng et al., 2019), and neutralization of extracellular histones by either complexation (Wildhagen et al., 2014; Silvestre-Roig et al., 2019; Schumski et al., 2021) or proteolytic cleavage (Chen et al., 2014) was shown to be beneficial in reducing the degree of tissue and organ damage.

Therefore, given the recorded involvement of neutrophil activation and potential contribution of elevated levels of histone H3 in severe COVID-19 patients, we aim to investigate the presence of extracellular histones and other NET-related components in the plasma of severe COVID-19 patients on admission and during their subsequent stay in the ICU.

## Method

### Collection of Patient Samples

This prospective observational study was approved by the Swedish Ethical Review Authority (No. 2020-01623) Informed consent was obtained by the patient or by next-of-kin if the patient were unable to make an informed decision. The Declaration of Helsinki and its subsequent revisions were followed. The protocol of the study was registered *a priori* (ClinicalTrials ID: NCT04316884). STROBE guidelines were followed for reporting. An initial description of this study could be found in Huckriede et al. (2021). All 117 patients admitted to the central intensive care unit at Uppsala University hospital during the first wave of the pandemic in 2020, with suspected COVID-19 infection, were screened for inclusion. The diagnosis for COVID-19 was performed by polymerase chain reaction (PCR) for SARS-CoV-2 in airway secretion. For a subset of patients, longitudinal plasma samples were available (n=54) taken on ICU days 1 and 4. Additionally, 11 ICU control patients from the same ICU ward, who were negative for COVID-19 screening and all underwent hyperthermic intraperitoneal chemotherapy (HIPEC) surgery, and 15 healthy volunteers were included. A first blood sample (in citrate buffer) was collected after patient admission to the ICU. Platelet poor plasma (PPP) samples were prepared by centrifugation (3000 RCF for 10 min at 4°C) of collected blood and supernatant was carefully removed and snap-frozen until used. Clinical data were recorded prospectively including medical history, medications, physiological data, level of organ support, and date of death. Simplified acute physiology score 3 (SAPS3) (Moreno et al., 2005), Sequential Organ Failure Assessment (SOFA) score (Vincent et al., 1996), and organ support data were collected as reported in the results. Demographical data was extracted from the patient records.

### Western Blot Method & Image Analysis

Plasma samples were analyzed for the presence of histone H3 *via* semi-quantitative Western blotting as previously described (Abrams et al., 2013; Wildhagen et al., 2015; Cheng et al., 2019). In brief, equal volumes of 10-fold diluted plasma patient samples were separated *via* SDS-PAGE gel electrophoresis (4-15%) always together with a known concentration standard range (0.01 – 0.04 µg) of purified calf thymus H3 (Roche, Basel, Switzerland) as shown in **Supplemental Figure 1**, and transferred to PVDF membranes (Bio-Rad Laboratories, Hemel Hempstead, UK) using semi-dry blotting. After blocking, the membranes were incubated with primary anti-histone H3 antibody (1:10.000 o/n at 4°C, sc-8654-R, Santa Cruz Biotechnology, Heidelberg, Germany), followed by a secondary biotin-conjugated IgG antibody (1:10.000 for 30 min at RT, ab97083, Abcam, Cambridge, UK) and a streptavidin-biotin/alkaline phosphatase complex (1:500 for 30 at RT, Vectastain ABC-Alkaline Phosphatase, Vector Laboratories, Burlingame, USA). Histone H3 bands were visualized by WesternBright ECL substrate (Advansta, San Jose, California, USA) and band densities were quantified by ImageStudioLite software, as compared to the known concentrations of purified H3, with a detection limit of 0.005 µg/ml.

### ELISA (NE, MPO, and MPO-cfDNA)

NE and MPO levels in plasma were determined by the ELISA technique using commercial kits from R&D systems (DuoSet ELISA, Bio-Techne, Minneapolis, USA) according to the manufacturer’s instructions. Dilution of plasma samples was 1:50 in 1% BSA reagent diluent for both markers. MPO-cfDNA levels in plasma were determined using the protocol as described by Kano et al. (2017), using an anti-MPO capture antibody (Merck Millipore Corp) in combination with the peroxidase labeled anti-DNA antibody (Roche Diagnostics, Indianapolis, Indiana, USA) to detect the complex of MPO-DNA in 1:4 diluted plasma.

### ALU-60 qPCR (cfDNA)

Eight times diluted plasma samples were analyzed for the presence of cell-free DNA (cfDNA) in COVID-19 positive plasma samples *via* ALU-60 qPCR (Breitbach et al., 2014; Leest et al., 2020). In short, plasma samples were diluted 8-fold in water to result in a final assay dilution of 40 times. The total reaction volume contains 5 µL TATAA Probe GrandMaster Mix/no ROX (TATAA Biocenter), 0.5 µL TATAA Alu-60 assay probes (TATAA Biocenter), 2.5 µL H2O and 2 µL of a sample, which was pipetted into a 96-well plate (Roche) and measured with a LightCycler 480 qPCR machine (Roche). The thermal cycling conditions started with a DNA-denaturation step at 95°C for 2 min, followed by 40 cycles of denaturation at 95°C for 5 s, annealing at 60°C for 10 s and extension at 60°C for 30 s. DNA concentrations were compared to a calibration range (from 1 to 300 ng/µL) using a purified and quantitated DNA standard.

### Data Analysis and Statistics

Graphpad Prism version 8 (Graphpad Software Inc., La Jolla, CA, USA) and SPSS Statistics version 26 (SPSS Inc., Chicago, IL, USA) were used for statistical analysis. Kolmogorov-Smirnov tests were employed to inspect the normality of data. Parametric data are presented as mean (SD) or geometric mean (95% CI) for log-transformed data unless stated. Non-parametric data are presented as median and interquartile range (IQR). The Kruskal Wallis test was used to analyze variance, Mann-Whitney U tests to test unpaired groups, Wilcoxon matched-pairs signed-rank to test paired groups, and the chi-square test for categorical parameters. Spearman’s rank-order test with a Bonferroni correction for multiple testing was used to calculate correlations. Univariate regression analysis was used to analyze associations between histone status and pathologic events. P-values were considered significant if p < 0.05 unless stated; Significance is indicated as *p <0.05, **p <0.01, ***p <0.001.

## Results

The presence of the individual NET-related markers histone H3, cfDNA, NE, MPO, and MPO-DNA complexes was assessed in citrated plasma of 117 SARS-COV-2 positive patients admitted to the ICU at Uppsala University Hospital, Sweden. Additionally, 11 non-SARS-CoV-2 ICU patients and 15 healthy volunteers were included as controls (**Supplementary Table 1**). The COVID-19 patients were 61 years old on average, and the majority (76%) were male. The mean arterial pressure (MAP), respiratory rate, and temperature were significantly increased compared to the control ICU group. Plasma samples were collected on admittance and subsequent days at the ICU department.

**Figure 1:**
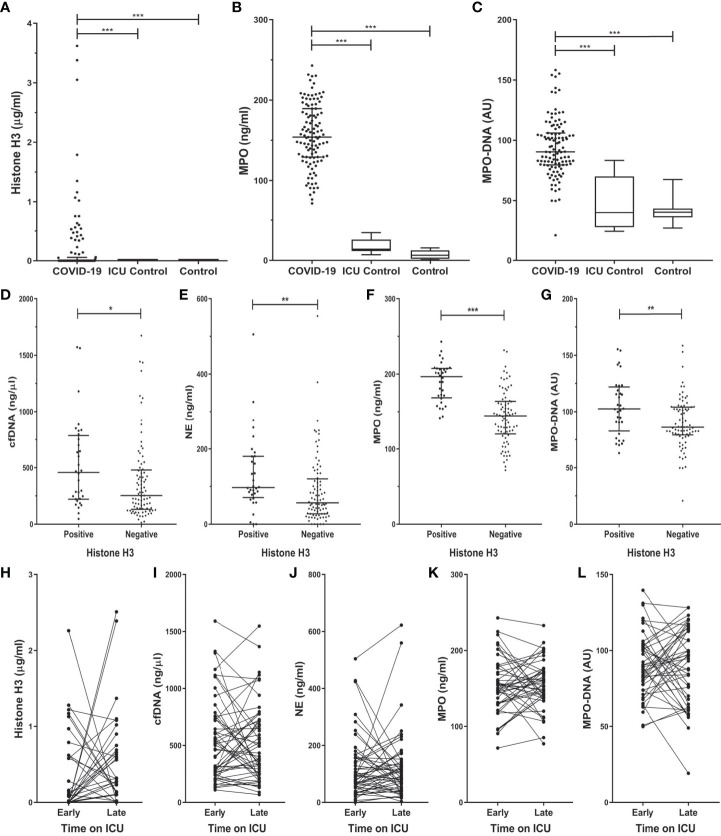
Histone H3 and NET components increased in COVID-19 ICU patients on admission **(A–C)**, are increased in COVID-19 histone H3 positive patients **(D–G)** and subsequent stay **(H–L)** at the ICU department. Plasma from COVID-19 (n=117), non-COVID-19 ICU patients (n=11), and healthy control (n=15) was tested for the presence, represented as median (IQR) or box and whisker plot, of histone H3 **(A)**, myeloperoxidase (MPO) **(B)**, and the MPO-DNA complex **(C)**. COVID-19 samples were compared to control groups with the Kruskal-Wallis test with Dunn’s post-hoc test. The level of cfDNA **(D)**, NE **(E)**, MPO **(F)**, MPO-DNA **(G)** in the COVID-19 histone H3 positive and negative groups. Samples were compared by Mann-Whitney U test. The level of histone H3 **(H)**, cfDNA **(I)**, NE **(J)**, MPO **(K)**, and MPO-DNA **(L)** of a subset of COVID-19 patients were followed in time on early (1-3) and late (≥ 4) days at the ICU department. Samples Early-Late were compared by Wilcoxon matched-pairs signed-rank test. P-values were considered significant if p < 0.05; *0.05, **0.01, ***0.001.

The levels of histone H3, MPO, and MPO-DNA complex (**Figures 1A–C**) were all significantly increased in COVID-19 patients on admission to the ICU as compared to both control groups. The level of histone H3 significantly correlated to MPO (r_s_ = 0.440; p <0.001), and MPO-DNA complex (r_s_ = 0.249; p = 0.009) in the COVID-19 patients.

On admission, histone H3 was present in 28% of the patients of which the median [IQR] concentration was 0.48 [0.16 – 0.95] µg/ml. No histone H3 was detected in either of the control groups. In COVID-19 patients with confirmed histone H3 presence on admission, the concentration of cfDNA, NE, MPO, and MPO-DNA complex was significantly increased compared to the histone H3 negative patients (**Figures 1D–G**).

For a subset of 54 COVID-19 patients, sequential samples were available of early (first 3 days) and late (4+ days) stay at the ICU department. No significant difference could be observed in any of the measured NET markers between the two time points (**Figures 1H–L**). However, the difference between early and late stay at the ICU correlated between histone H3 and NE (r_s_ = 0.308; p = 0.035), and between histone H3 and the MPO-DNA complex (r_s_ = 0.460; p = 0.001).

**Figure 2:**
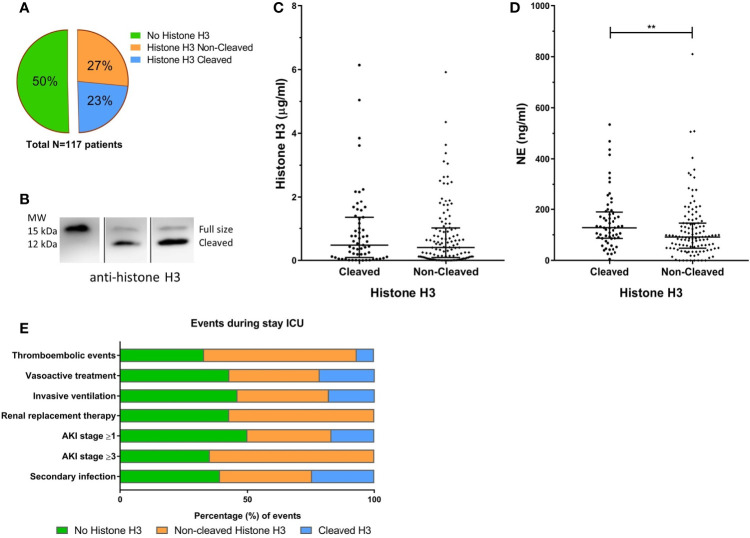
Histone H3 cleavage in COVID-19 patients. The fraction of patients with detectable cleaved or non-cleaved histone H3 **(A)**. Western blot analysis using a polyclonal anti-histone H3 antibody allowed detection of proteolytic cleavage of histone H3, distinguishing between full-sized histone H3 (15 kDa) and a histone H3 fragment (12 kDa) **(B)**. The concentration of histone H3 **(C)** or NE **(D)** in cleaved or non-cleaved histone H3 positive samples. The percentage (%) for the positive amount of significantly different clinical events during the stay at the ICU **(E)**. Acute kidney injury (AKI). P-values were considered significant if p < 0.05; *0.05, **0.01, ***0.001.

Detectable levels of histone H3 were found in 50% of the COVID-19 ICU patients at least once during their stay at the ICU (**Figure 2A**) with a median concentration of 0.43 [0.09 – 1.15] µg/ml. As we observed proteolytic cleavage of histone H3 in patient plasma, the apparent molecular weight was analyzed to distinguish the full-sized from cleaved histone H3 (**Figure 2B**). In 23% of the COVID-19 patients, histone H3 was fully or partially cleaved, indicating processing of histones in COVID-19 patients. Median total histone concentration was similar in samples that show cleavage and those not cleaved (**Figure 2C**). The level of NE, a serine protease able to cleave histones (Papayannopoulos et al., 2010), was significantly increased in samples that contained cleaved histone H3 (172.9 vs. 92.1 ng/ml; p = 0.006) (**Figure 2D**).

The presence of any form of histone H3 during the stay in the ICU was associated with an increased incidence of thromboembolic events, and the presence of a secondary infection (**Figure 2E**). Furthermore, the very presence of non-cleaved histone H3 is significantly associated with the increased occurrence of a thromboembolic event, the need for vasoactive treatment, invasive ventilation, and the development of AKI and the subsequent need for renal replacement therapy compared to patients with cleaved histones (**Supplemental Table 2**). The status of histone H3 or any of the other NET components was not associated with an increased risk of 30-day mortality.

## Discussion and Conclusion

The presence upon admission and persistently elevated levels of the NET-related markers histone H3, cfDNA, NE, MPO, and the MPO-DNA complex indicate activation of neutrophils and the formation of NETs in the plasma of COVID-19 patients admitted to the ICU. The harmful effect of extracellular histones in COVID-19 disease progression is further suggested by findings of histone H3 in 50% of critically ill COVID-19 patients and their association with adverse outcomes.

The presence of increased levels of NET components after viral infection is not limited to SARS-COV-2, as increased levels were observed after infection with influenza A, dengue, HIV-1, and respiratory syncytial virus (RSV). Therefore care should be taken not to interpret our data as being selective for COVID-19 patients. Furthermore, all COVID-19 patients studied were severely ill, making it hard to discriminate the clinical outcomes in a homogenously affected population. Of note, the control groups used here were included to serve the purpose of internal data validation and to indicate the abnormality of the findings in the COVID-19 group. The control ICU group is relatively small and was uniformly admitted for HIPEC surgery, which limits the ability to extrapolate our findings to other ICU populations. Since this ICU control group and the healthy individuals did not present with infectious disease, care should be taken to not interpret our data as being characteristic for exclusively COVID-19 patients. Rather, they may represent characteristics of a more general type of critically infected patient. ICU admission of the patients in the present study was on average 10 days after the onset of COVID-19 related symptoms. Therefore, the present data do not provide exact timing of the onset of NETosis during COVID-19. Consequently, the exact relation between NETosis and thromboembolic events or secondary infection is unknown, limiting the ability to distinguish between event-dependent changes in NET components. However, the presence of histone H3 suggests a role of histone-mediated damage on the development of a thrombotic phenotype in COVID-19. Increased levels of histone during sepsis or trauma (Hoeksema et al., 2016) or after *in vivo* administration of histones (Xu et al., 2009) have previously been shown to promote coagulopathy and thrombosis. In addition, as the incidence of adverse events is increased in the non-cleaved histone H3 group, *in vivo* proteolytic cleavage of histones hypothetically represents a regulatory mechanism to limit histone-mediated processes. Several studies (Busch et al., 2020; Ng et al., 2020) have reported the elevated levels of wild type and citrullinated histone H3 in COVID-19 patients, we are the first to have identified high levels of cleaved histone H3 among a sizeable ICU population of COVID-19 patients. While the nature of the peptidase responsible for the cleavage has not been revealed, NE is a likely candidate, considering the correlation between the observed cleavage and NE levels. Moreover, since the epitope of the antibody used for histone detection resides in the C-terminus of histone H3, a cleavage near the N-terminus of histone H3 could explain the observed cleavage patterns. The increased secondary infection at the ICU indicates the additional role of opportunistic pathogens in the disease progression of COVID-19.

In conclusion, the data of this study support the validity of treatments that aim to reduce NET formation and additionally underscore the role of histones in COVID-19. Therefore, besides suppression of NET formation in general, more targeted therapies focusing on the neutralization of the harmful effects of histones should be considered as treatment options for severe COVID-19 patients (Barnes et al., 2020; Cicco et al., 2020).

## Data Availability Statement

The original contributions presented in the study are included in the article/**Supplementary Material**. Further inquiries can be directed to the corresponding authors.

## Ethics Statement

The study was approved by the Swedish National Ethical Review Agency (EPM; No. 2020-01623). Informed consent was obtained from the patient, or next of kin if the patient was unable give consent. The Declaration of Helsinki and its subsequent revisions were followed. The protocol of the study was registered (ClinicalTrials ID: NCT04316884). The patients/participants provided their written informed consent to participate in this study.

## Author Contributions

GN was involved in the conception and design of the study. RF, MH, and ML participated in data collection, analysis and interpretation. JH and FV performed and analyzed the experiments. KW and CR contributed to supervision and data analysis and provided intellectual input. GN and JH drafted the manuscript, GN provided funding, performed experiments, and analyzed data. All authors contributed to the article and approved the submitted version.

## Funding

SciLifeLab/KAW national COVID-19 research program project grant (KAW 2020.0182 and KAW 2020.0241) and from the Swedish Heart-Lung Foundation (20210089) to MH. The Swedish Research Council (2014-02569 and 2014-07606) and The Swedish Kidney Foundation (F2020-0054) to RF. Netherlands Thrombosis Foundation (2016_01) to GN

## Conflict of Interest

CR, RF and GN are scientific advisor at Matisse Pharmaceuticals B.V.

The remaining authors declare that the research was conducted in the absence of any commercial or financial relationships that could be construed as a potential conflict of interest.

## Publisher’s Note

All claims expressed in this article are solely those of the authors and do not necessarily represent those of their affiliated organizations, or those of the publisher, the editors and the reviewers. Any product that may be evaluated in this article, or claim that may be made by its manufacturer, is not guaranteed or endorsed by the publisher.

## Supplementary Material

Supplementary Figure 1 Overview of histone H3 western blot detection method. On the left western blot, the molecular weight marker is followed by the histone H3 standard, with a molecular weight around 15 kDa, followed by 10 patient samples. Sample 3 – 6 are examples of negative histone H3 samples, samples 2 and 7 are examples of positive uncleaved H3 samples with one band at 15 kDa. On the right western blot, the protein standard is followed by 10 patient samples. In this blot, samples 1 and 6 are an example of a positive cleaved histone H3 sample (with one band at 12 kDa). Sample 1 shows also both a faint uncleaved and cleaved histone H3 (bands at 15 and 12 kDa).

Supplementary Table 1 Demographic and baseline characteristics of 128 patients on admission to the Intensive care unit. Values are represented as median (IQR) or n (%).

Supplementary Table 2 Events during stay at the ICU for COVID-19 ICU patients, divided by histone H3 status. The number (%) of events for each group is calculated based on the group column total.
