# Supplementary figures and images for: Histone H3 Cleavage in Severe COVID-19 ICU Patients
Source: Front Cell Infect Microbiol. 2021 Sep 10;11:694186. doi: 10.3389/fcimb.2021.694186 (PMC8461091; doi:10.3389/fcimb.2021.694186)

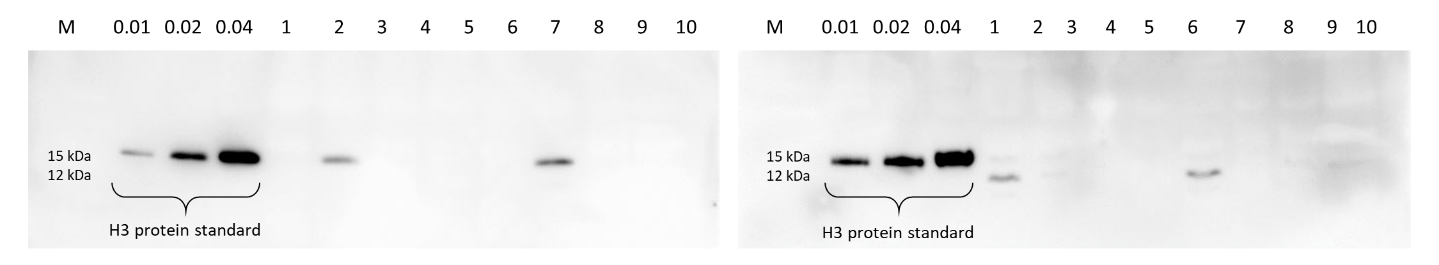


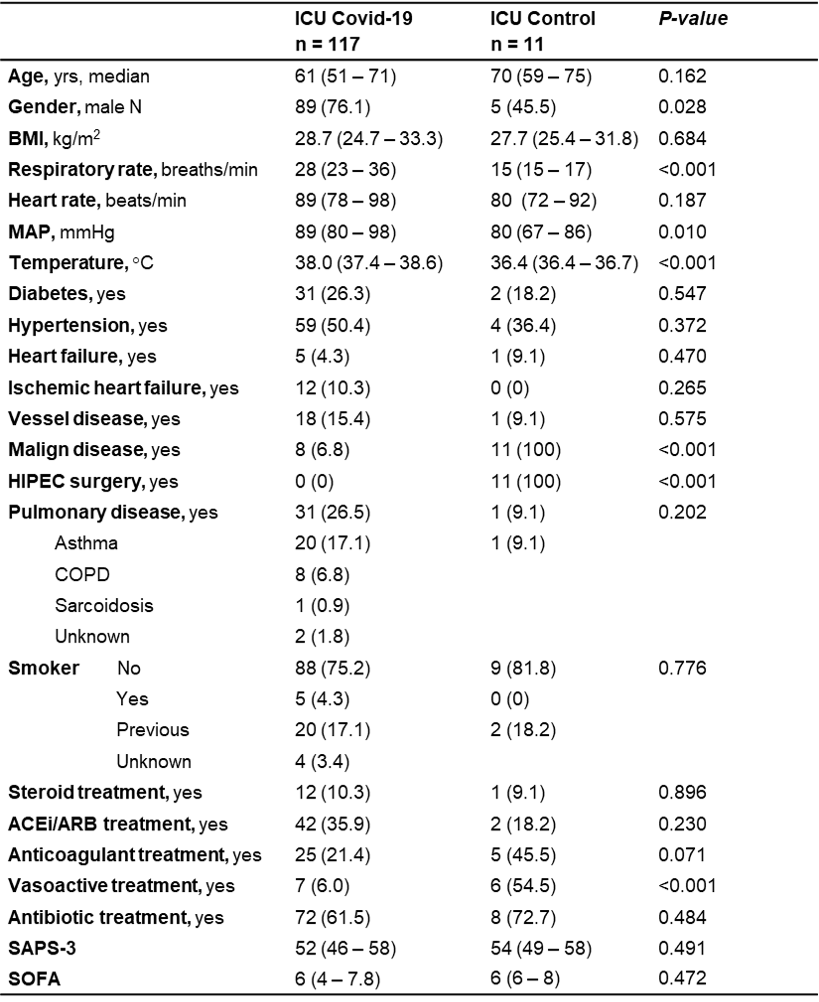

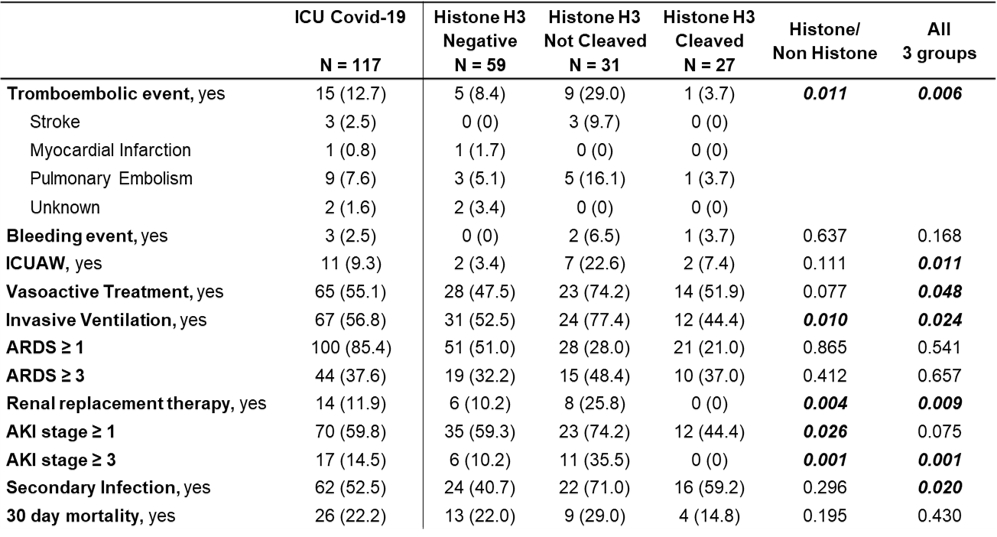

Supplement: Supplementary Figure 1 — Overview of histone H3 western blot detection method. On the left western blot, the molecular weight marker is followed by the histone H3 standard, with a molecular weight around 15 kDa, followed by 10 patient samples. Sample 3 – 6 are examples of negative histone H3 samples, samples 2 and 7 are examples of positive uncleaved H3 samples with one band at 15 kDa. On the right western blot, the protein standard is followed by 10 patient samples. In this blot, samples 1 and 6 are an example of a positive cleaved histone H3 sample (with one band at 12 kDa). Sample 1 shows also both a faint uncleaved and cleaved histone H3 (bands at 15 and 12 kDa). [file Table_1.docx]
